# Impact of telehealth on health care resource utilization during the COVID-19 pandemic

**DOI:** 10.2217/cer-2021-0242

**Published:** 2022-01-18

**Authors:** Prachi Arora, Darshan Mehta, Jane Ha

**Affiliations:** ^1^College of Pharmacy & Health Sciences, Butler University, 4600 Sunset Ave, Indianapolis, IN 46208, USA; ^2^Sunovion Pharmaceuticals Inc., 84 Waterford Drive, Marlborough, MA 01752, USA

**Keywords:** COVID-19, healthcare resource utilization, telehealth

## Abstract

**Aim:** To assess healthcare utilization (HCRU) among patients with incident telehealth visit during the COVID-19 pandemic. **Materials & methods:** Retrospective pre-post analyses was conducted using HealthJump data. Adults continuously enrolled with an incident telehealth visit between Feb and April 2020 were identified. Demographics, clinical characteristics, proportion of patients with ≥1 HCRU visits and post-index trends in HCRU were analyzed. **Results:** Sample constituted 2799 patients, 60.34% female and 46.23% white with mean age 59.70. Significant increase in patients with outpatient visits (5.36%, p < 0.005; only established), non-face-to-face visits (99.50%, p < 0.005) and prescription use (12.86%, p < 0.005) was reported. **Conclusion:** Among patients utilizing telehealth during COVID-19 pandemic, HCRU changed significantly. Better deployment policies and adoption techniques of telehealth could potentially act as a strong tool to revolutionize the healthcare delivery, with or without the pandemic.

Social distancing became an integral part of our daily lives during the COVID-19 pandemic, which hindered the standard course of healthcare delivery for patients, clinicians and policy-makers [1,2]. Globally, telehealth emerged as a solution to support timely access to care and expand the delivery of healthcare [3].

According to a report by CDC, telehealth encounters were 154% higher during week 13 of 2020 compared to the same time frame in 2019 [4]. Within a month’s duration in 2020, the use of digital health by Oregon Health & Science University increased dramatically from 1100 visits in February to 13,000 visits in March 2020 [5]. Additionally, a recent study reported the highest peak in telehealth visits during April 2020 as being 78 times higher than February 2020 [6]. That being said, a number of determinants impeded the rapid adoption of telehealth by physicians and patients during the COVID-19 including human (e.g., patient willingness), infrastructure (e.g., poor internet connection, data confidentiality) and institutional (technical support, availability of funds) factors [7].

To curtail the growing burden on healthcare and help meet the increasing needs of telehealth services during the pandemic, Centers for Medicare & Medicaid Services (CMS) repurposed some of the current procedural terminology (CPT) codes [8]. CMS also adopted major legislative and regulatory changes to reimbursements in March 2020 such that Medicare would pay for office, hospital and other visits furnished via telehealth across the country including the patient’s place of residence [9]. Other payers, such as United Healthcare and Cigna, also updated their policies to continue payment for telehealth services through 2021 [10,11].

While telehealth visits rose spontaneously during the pandemic, early studies report a decrease in healthcare utilization (HCRU) including inpatient visits, emergency department visits and outpatient visits [12–15]. Some of the reasons for the decrease in the use of traditional healthcare could be the over-burdened health systems, a patient’s fear of contracting the COVID-19 infection during in-person visit, and CDC’s recommendation to delay elective care [12–15].

With some evidence suggesting that traditional HCRU decreased during the pandemic, little is known about the extent and nature of the telehealth visits. Around strong indications that telehealth would continue to be a major contributor to health care delivery post-COVID-19, it is necessary to evaluate the role of telehealth in provision of care during the current course of the ongoing COVID-19 pandemic [6,16]. Since many patients were forced to seek care remotely under the telehealth provisions during the COVID-19 pandemic, this study intends to assess the HCRU among the naïve users of telehealth with an incident telehealth encounter during the pandemic.

The objective of this study was to assess the HCRU among the patients who received care via incident telehealth visits during the COVID-19 pandemic.

## Materials & methods

### Data

The study used the medical care utilization data from HealthJump electronic medical records extracted from 1 August 2019 to 30 June 2021, inclusively. The database is pro-bono, cross-industry initiative, composed of institutions donating de‐identified data for COVID-19 research.

### Study design

**Figure 1.**
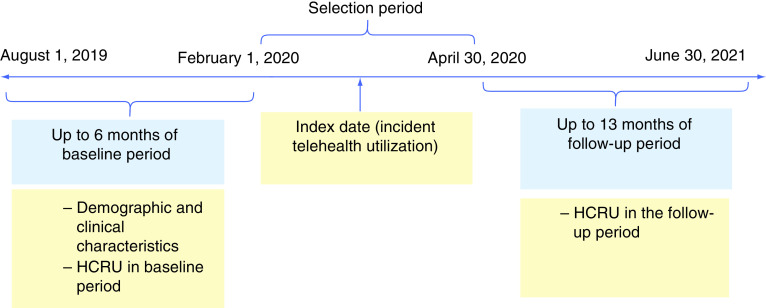
Study design. HCRU: Healthcare utilization

This was a retrospective pre–post cohort study design. The baseline period was defined as the 6-month period prior to the index date. The follow-up period was defined as the 13-month period following the index date, based on the availability of the data. The index date was the date of the first claim for telehealth visit during the index period (1 Feb–30 April 2020). Figure 1 shows an overview of the study design.

### Patient population

Patients with an incident telehealth visit during the index period were identified using keywords like ‘tel’, ‘web’ or ‘phone’ obtained from the description of the encounter type. Among those with an incident telehealth visit (n = 227,550), those patients who had a CPT code for any HCRU as defined by the CMS [8], within 7 days of the telehealth encounter were identified. Index date for a patient was defined as the telehealth encounter date with a CPT code within 7 days. Patients with any record of telehealth visits during the baseline period were excluded from the analyses. Patients were included if they were continuously enrolled during the study duration, in other words, having a medical and a pharmacy claim in the baseline and follow-up period. Patients were included if they were 18 years or older on the index date and US residents. A subgroup analysis was also conducted among patients with the top five most prevalent comorbid conditions (or high comorbidity).

### Study measures

During the baseline period, demographic and clinical characteristics such as age, gender, race, ethnicity, location and the diagnosis on the index date, were assessed for the overall sample and the high comorbidity subgroup. During the baseline and follow-up period, proportion of patients with ≥1 HCRU visits in each of the following categories were evaluated for the overall sample and the high comorbidity subgroup: office or outpatient (OP) visits including new or established patients; inpatient or hospital observation (IP) visits; consultations; mental health; preventive medicine (PM); non-face-to-face (F2F) services; other services (including emergency department visits, critical care, nursing facility and home services) ; and prescriptions. Each HCRU was identified using the CPT codes displayed in the appendix.

### Statistical analyses

Summary statistics were calculated for the demographic and clinical characteristics of the overall sample and the high comorbidity subgroup. The proportion of patients with ≥1 HCRU was calculated for the overall sample and the high comorbidity subgroup during the pre-index and the post-index period. Changes in the proportion of patients with ≥1 HCRU were compared using McNemar’s test. Post-index monthly trends in the number of claims for each HCRU category were also assessed for the overall sample and displayed in a graph format. A p-value of ≤0.05 was considered statistically significant. All the analysis was conducted using SQL and SAS statistical software version 9.4.

## Results

**Figure 2.**
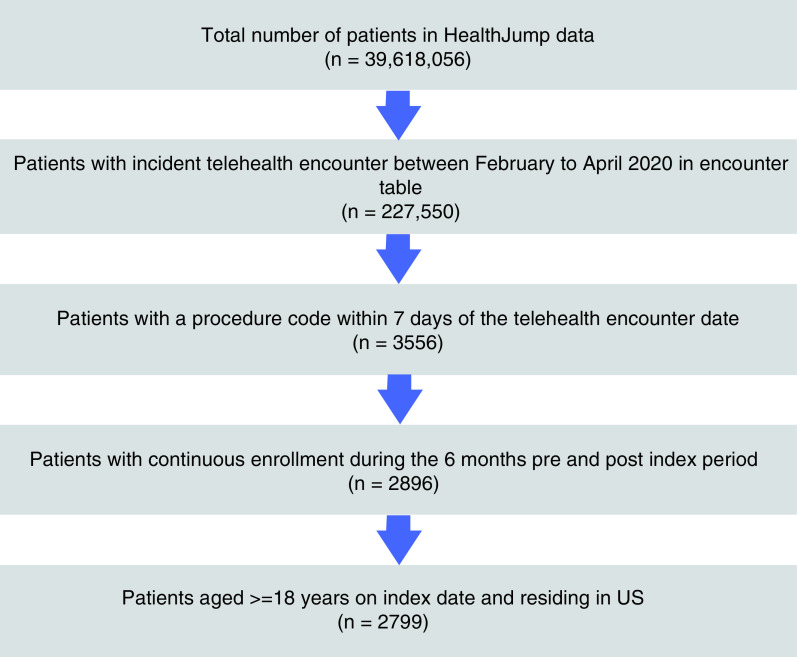
Sample selection.

Of the 39,618,056 patients included in the HealthJump databases, 227,550 had an incident telehealth visit during February–April 2020, and a sample of 2799 patients met the inclusion and exclusion criteria. The details of the sample attrition at each step of the inclusion-exclusion criteria are summarized in Figure 2.

Table 1 summarizes the demographic and clinical characteristics of the overall sample and the high comorbidity subgroup. Overall sample constituted of 2799 patients with a mean (SD) age of 59.70 (16.70), 60.3% female and 46.2% white. About 75% of the overall sample population resided in the following states: West Virginia, California, Louisiana, Mississippi and New Jersey. The top diagnosis associated with the index telehealth visit in the sample population included hypertension, hyperlipidemia, gastro-esophageal reflux disease, low back pain and Type 2 diabetes mellitus (results not shown). Among the subgroup of patients with top five diagnosis or high comorbidity, the mean age was higher (63.24 years). Other baseline characteristics of the high comorbidity subgroup were similar to the overall sample.

**Table 1.** Baseline demographic and clinical characteristics of the overall sample and high comorbidity patient subgroup.

Demographic variables	Overall sample (n = 2799)		High comorbidity patient subgroup (n = 1535)	
	Frequency	Percentages	Frequency	Percentages
Age, mean (standard deviation)	59.70 (16.70)		63.24 (14.55)	
Gender:				
– Female	1689	60.34%	907	59.09%
– Male	1110	39.66%	628	40.91%
Race:				
– White	1294	46.23%	788	51.34%
– Black or African–American	305	10.90%	141	9.19%
– American–Indian or Alaska native	12	0.43%	6	0.39%
– Asian	10	0.36%	1	0.07%
– Native Hawaiian or other Pacific Islander	2	0.07%	1	0.07%
– Unknown	636	22.72%	219	14.27%
– Frequency missing	540	19.29%	379	24.69%
Ethnicity:				
– Not Hispanic or Latino	1963	70.13%	1233	80.33%
– Hispanic or Latino	87	3.11%	31	2.02%
– Unknown	338	12.08%	126	8.21%
– Frequency missing	411	14.68%	145	9.45%
Location (top five states):				
– West Virginia	684	24.44%	549	35.77%
– California	507	18.11%	197	12.83%
– Louisiana	484	17.29%	236	15.37%
– Mississippi	260	9.29%	212	13.81%
– New Jersey	162	5.79%	66	4.3%

Table 2 summarizes the results of the pre–post comparisons for the overall sample and the high comorbidity subgroup of patients. Compared to the pre-index period, there was a significant increase in the proportion of established patients with OP visits in the post-index period for the overall sample (80.99 vs 86.35%; p-value < 0.05). As shown in Table 2, the proportion of new patients with OP visits decreased significantly for the overall sample (43.09 vs 6.18%; p-value < 0.05) and the high comorbidity subgroup (40.72 vs 2.45%; p-value < 0.05).

**Table 2.** Comparison of the healthcare utilization categories pre-post the telehealth index visit for overall sample and high comorbidity patient subgroup.

Utilization variables	Overall sample (n = 2799)				High comorbidity patient subgroup (n = 1535)			
	Pre	Post	Diff	p-value	Pre	Post	Diff	p-value
Office visit or outpatient visits:	88.96%	87.42%	‐1.54%	0.1023	92.64%	90.23%	‐2.41%	0.0262^†^
– New patient office or other outpatient services	43.09%	6.18%	‐36.91%	<0.05^†^	40.72%	2.35%	‐38.37%	<0.05^†^
– Established patient office or other outpatient services	80.99%	86.35%	5.36%	<0.05^†^	87.75%	89.71%	1.96%	0.1189
Inpatient or hospital observation visits:	4.57%	1.82%	‐2.75%	0.3019	3.97%	2.08%	‐1.89%	0.6078
– Inpatient services	4.54%	1.71%	‐2.83%	0.2895	3.91%	1.89%	‐2.02%	0.5827
– Hospital observation services	0.32%	0.11%	‐0.21%	0.9467	0.26%	0.20%	‐0.06%	1.0000
Consultation	4.36%	1.39%	‐2.97%	0.2661	3.78%	1.24%	‐2.54%	0.4873
Mental health	0.39%	0.43%	0.04%	1.0000	0.26%	0.46%	0.20%	0.9712
Preventive medicine:	18.40%	27.01%	8.61%	0.0003^†^	17.46%	21.63%	4.17%	0.2049
– New patient preventive medicine services	2.22%	0.18%	‐2.04%	0.4515	1.11%	0.13%	‐0.98%	0.7999
– Established patient preventive medicine services	12.58%	9.72%	‐2.86%	0.2626	11.60%	9.51%	‐2.09%	0.5541
– Counselling risk factor reduction and behavior change services	3.00%	2.07%	‐0.93%	0.735	3.65%	2.08%	‐1.57%	0.6736
– Other preventive medicine services	0.04%	14.58%	14.54%	<0.05^†^	0.00%	8.60%	8.60%	0.0156^†^
Non-face-to-face:	0.50%	100.00%	99.50%	<0.05^†^	0.46%	100.00%	99.54%	<0.05^†^
– Non-face-to-face-digital	0.04%	14.58%	14.54%	<0.05^†^	0.00%	8.60%	8.60%	0.0156^†^
– Non-face-to-face-telephone	0.46%	85.49%	85.03%	<0.05^†^	0.46%	91.40%	90.94%	<0.05^†^
– Inter-professional telephone/internet/electronic health record consultations	0.00%	0.07%	0.07%	0.9893	0.00%	0.07%	0.07%	1.0000
– Digitally stored data & remote physiological monitoring services	0.00%	0.14%	0.14%	0.968	0.00%	0.00%	0.00%	1.0000
– Remote physiologic monitoring treatment management services	0.00%	0.07%	0.07%	0.9893	0.00%	0.00%	0.00%	1.0000
Other visits (including emergency department, critical care, nursing facility, home services)	0.57%	0.14%	‐0.43%	0.8829	0.52%	0.20%	‐0.32%	0.9423
Prescriptions	80.67%	93.53%	12.86%	<0.05^†^	87.17%	95.57%	8.40%	<0.05^†^

† Significant at p ≤ 0.05.

Diff: Difference in the percentages of patients utilizing healthcare in the post vs pre-index period.

Compared to the pre-index period, there was a decrease in the proportion of patients with IP visits and consultations, but the results were not statistically significant (Table 2). The proportion of patients with PM increased overall from the pre vs post period; however, within the subcategories of PM, the proportion of new and established patients using PM decreased post-index (Table 2).

The proportion of patients with non-F2F visits increased from pre to post-index period, with around 85% of the claims under the non-F2F telephone subcategory in the post-index period (Table 2). There was a decrease in the proportion of patients under the other visits category which included emergency department visits, critical care, nursing facility and home services for the overall sample and high comorbidity subgroup, though results were not statistically significant (Table 2).

Compared to the pre-index period, the number of prescriptions rose tremendously in the post-index period for the overall sample (37,326 vs 51,179) and high comorbidity subgroup (23,750 vs 30,761). As shown in Table 2, the proportion of patients with the prescription claims increased significantly from pre to post index for the overall sample (80.67 vs 93.53%, p-value < 0.05) and high comorbidity subgroup (87.17 vs 95.57%; p-value < 0.05).

**Figure 3.**
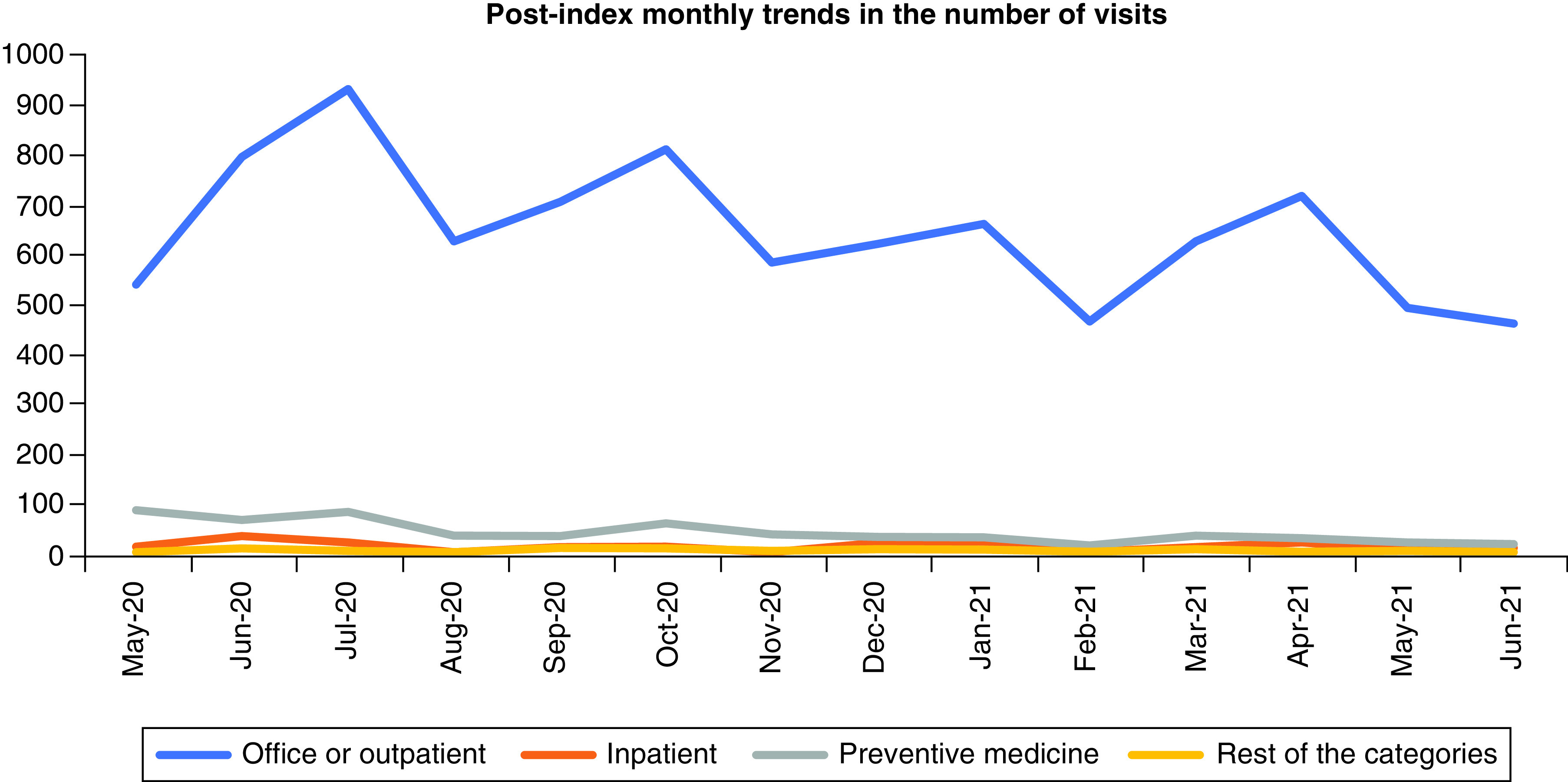
Post-index monthly trends in the number of office or outpatient, inpatient, preventive medicine and other visits for the overall population. Rest of the categories include visits, such as emergency department, critical care, nursing facility, home services, consultation visits and mental health visits.

**Figure 4.**
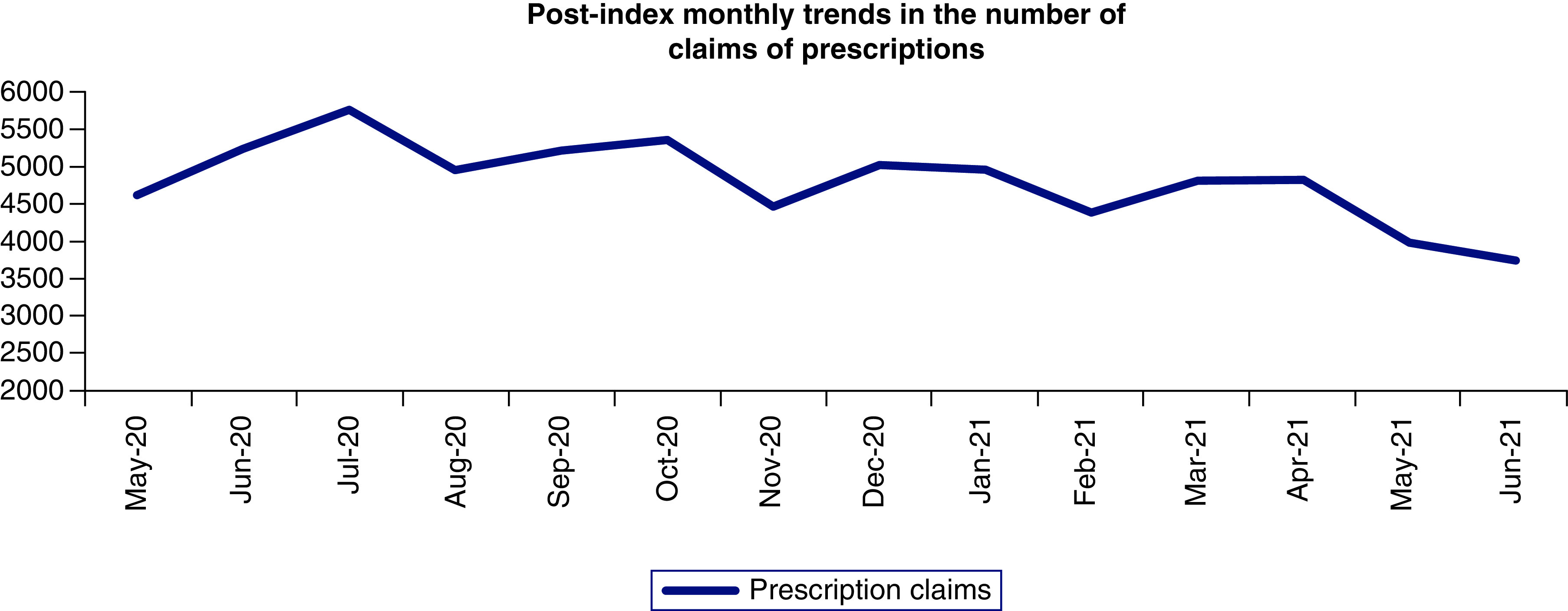
Post-index monthly trends in the number of claims of prescriptions for the overall population.

The post-index monthly trends in the OP, IP, PM and rest of the categories are shown in Figure 3 and prescription use in Figure 4. Among all the utilization variables, OP visits and prescription claims were the highest for the overall sample post index (Figures 3&4). The trends in the OP visits and prescription claims follow a similar pattern from May 2020 to June 2021, with a gradual increase/decrease observed throughout the post index (Figures 3&4). The trends in the number of IP and PM visits were low throughout the study duration (Figure 3).

## Discussion

To the author’s knowledge this is a first-of-a-kind study conducted on the patients who were naïve to telehealth before the pandemic and had their first telehealth visit during the index period. This study is also novel in assessing the changes in the different categories of HCRU delivered via telecare during the pandemic.

Our study indicates that there was a significant increase in the proportion of patients with office/OP visits (established patients only) and prescription claims. However, a significant decrease was seen in the proportion of patients (both new and established) utilizing PM from pre to post-index period. These results suggest that patients with telehealth knowledge and access might have considered deferring the use of PM during the pandemic; however, continued the OP visits and prescription use. A past study [17], in concordance with our study, reported dramatic reductions in the use of preventive and elective care during the first 2 months of COVID-19 pandemic and indicated that foregoing or deferring preventive care could add to the cost burden of the healthcare system in the long run.

Our study also assessed the monthly trends in the HCRU post the index date for the incident telehealth users (Figures 3 & 4). Patients with an incident telehealth use during the peak of pandemic (February–April) consistently had substantial number of OP visits and prescription claims post the index date till June 2021 (Figures 3 & 4). Continued OP visits and prescription use among the patient population during the pandemic could mean that physicians were probably trying to effectively use telehealth as a tool and offset the burden caused by the deferred PM or other care. However, it could also translate into a concern of over or misprescribing of certain healthcare resources. An important result of our study was the dramatic increase in the number of prescription claims and the proportion of patients using prescription drugs pre-post index date. Around 94–96% of all the patients had at least one prescription fill in the post-index period for the overall sample and the high comorbidity subgroup. Such a significant increase in the prescription use during this study’s duration could be due to physicians handing out newer prescriptions or overprescribing to the patients post the index telehealth visit. Due to the limitations of our dataset, a comprehensive list of all the prescription drugs and the date of fill could not be obtained. Future research is needed to evaluate the long-term repercussions of adopting telehealth specifically on the prescriptions use and misuse, probably with a focus on opioids.

The patient population of our study was older and sicker and most encounters during the study period were reportedly for conditions not related to COVID-19. The top diagnoses associated with incident telehealth visits in our study were hypertension, hyperlipidemia, gastro-esophageal reflux disease, low back pain and Type 2 diabetes mellitus. Similar patient characteristics were reported by another published study conducted using the MarketScan and Medicare Supplemental Research Databases; however, the study was limited in the extent of healthcare outcomes assessed [18].

The results of our study which suggest a decrease in the OP, IP, consultations and other visits (including emergency department, critical care, nursing facility, home services) and an increase in the non-F2F visits, are in concordance with a recent study published by Xu *et al.* conducted using a large integrated health care system in the USA [19]. Our study was conducted using the HealthJump database, which is a collection of electronic medical records from different health systems in the USA; and therefore, is diverse and more representative of the US population.

Additionally, the HCRU outcomes evaluated in our study were categorized by the CPT codes defined by the CMS [8], thus identifying the relevant patient population with incident telehealth more closely and accurately. Our study also reports changes in the utilization among a subcohort of higher risk patients with high comorbidity. Similar trends across both the samples of patient populations suggest that the impact of telehealth on the healthcare system could be driven more by the practitioners, as opposed to the needs of the patients.

The value of telehealth cannot be overemphasized. With continued pressure on the medical systems to reduce the cost, telehealth could be an effective tool to reduce healthcare delivery cost without compromising the quality of care. During the COVID-19 pandemic, telehealth served as a powerful alternative to the usual care since it eliminated physical contact, reduced travel, decreased wait times and improved patient’s access to care and medication adherence [20–24]. Furthermore, telehealth could also be beneficial in disease prevention, treatment guidance, remote training and consulting of medical staff, with or without the pandemic [25]. To deploy and adopt telehealth technology across the nation, improvements in organizational, technological and social constructs of the system are required, for example, increasing user awareness, improving data security and better training for physicians and medical staff [24].

Countries, such as Denmark, Australia and UK, have also facilitated wider adoption of telehealth to contain the pandemic as well as treat the non-COVID patients remotely [26]. One such vulnerable population which has been adversely impacted by the pandemic are mental health patients [27]. Coping with the loss of family, illness of self/family and financial stress, while being isolated at home or hospital can lead to anxiety in anyone but can be worse for the those with pre-existing mental health condition [27]. Future research should focus on assessing the mental health utilization via telehealth technology during the COVID-19 pandemic and evaluate the impact of reimbursement policies on the tele mental health utilization.

There are certain limitations of this study. The sample sizes and the patient distribution by each state was driven by the source of data, contributed for this pro-bono research. Hence, the external validity of the results might be limited. A telehealth visit was identified using the relevant CPT codes. However, these codes were repurposed during the time of COVID-19 and hence, may not represent an actual telehealth visit. The researchers of this study tried to mitigate this limitation by including encounter type as an additional variable to identify a televisit.

## Conclusion

Patients accessing care via telehealth during the COVID-19 pandemic experienced a drastic shift in the HCRU, especially in OP visits, PM and prescription use. The use of preventive services declined whereas patients with OP visits and prescription use increased after April 2020. Telehealth as a concept has existed for long; however, policies around its coverage or reimbursement are novel and have emerged due to the COVID-19 social distancing regulations. The results of our study emphasize the importance of identifying loopholes in the access-to-care and further guiding the policies for sustaining telehealth as a viable alternative, now and in the future. With or without a pandemic, telehealth could potentially act as a strong tool to revolutionize the healthcare delivery system everywhere.

**Summary points**

- Telehealth as a concept has existed for long; however, policies around its coverage or reimbursement are novel and have emerged due to the COVID-19 social distancing regulations.
- There was a significant increase in the proportion of patients with office or OP visits (established only) and prescription use from pre vs. post-index period.
- There was a significant decrease in the proportion of patients utilizing preventive medicine (both new and established) from pre vs. post-index period.
- Telehealth could potentially act as a strong tool to revolutionize the healthcare delivery system everywhere, with or without the pandemic. However, more research is warranted to evaluate the long-term repercussions of delivering care via telehealth.
